# Using serious mobile games to improve health literacy in rural Sub-Saharan Africa: A literature review

**DOI:** 10.3389/fpubh.2022.768252

**Published:** 2022-11-17

**Authors:** Ismaila Ouedraogo, Borlli Michel Jonas Some, Kiemute Oyibo, Roland Benedikter, Gayo Diallo

**Affiliations:** ^1^Bordeaux Population Health INSERM–U1219–Univ. Bordeaux, Bordeaux, France; ^2^Ecole Supérieure D'Informatique (ESI), Université Nazi Boni, Bobo Dioulasso, Burkina Faso; ^3^Electrical Engineering and Computer Science, York University, Toronto, ON, Canada; ^4^Center for Advanced Studies Eurac Research, Bozen/Bolzano, Italy

**Keywords:** health literacy, serious game, mobile technology, rural areas, Africa

## Abstract

The African gaming industry is beginning to flourish as a result of a rise in the availability of inexpensive phones and the number of mobile phone subscribers. It has enabled the development and implementation of mobile serious games to promote healthy behavior change in rural communities. This paper examines the use of mobile serious games in healthcare education, with a particular focus on those designed to increase health literacy in rural Africa. Identifying and addressing the design challenges and issues faced by people living in rural African communities through the use of persuasive mobile games can promote behavior change among these underserved communities. We used PubMed, Scopus, Google Scholar and manual search to identify relevant studies published from 2011 to July 2021. The literature review highlights how the identified challenges affect the implementation of persuasive strategies, suggests design solutions for overcoming them, and discusses how persuasive games can be tailored to suit the target rural African populations. Some of the identified challenges are technical in nature (e.g., access to electricity and internet connectivity), while others are not (e.g., language diversity and low literacy). As the number of serious games for healthcare education and awareness continues to increase, it is essential for the successful implementation of inclusive mobile health technologies in rural Africa to identify and address the specific challenges faced by underserved populations such as rural African communities.

## Introduction

Most of the United Nations (UN) Agenda 2030 depends on improving people's health and life expectancy (1). Citizens must be empowered through health literacy activities so that they are better able to acquire, comprehend, and use health-related information to make informed decisions (1). Many experts believe that digital technologies can deliver positive and better healthcare to Sub-Saharan Africa (SSA) (2). The ubiquitous nature of mobile technology and handheld devices, along with the growing popularity of games among people of all ages and ethnicities (3, 4), has made the implementation and delivery of serious mobile games a widespread practice. Due to the motivating pull that these games provide, they are becoming the ideal medium for delivering persuasive content designed to inspire behavior change. Serious games can motivate behavior change subtly while the player is having fun (3). RightWay Café for instance is an example of games intended to promote a nutritious and healthy diet. Research showed that the game was effective in teaching nutrition and weight management knowledge and increasing participants' perceived self-efficacy and the perceived benefits of healthy eating. Participants in the treatment group had higher self-efficacy than those in the control group after 1 month (4).

The recent spread of cheaper Android phones across the African tech market has resulted in a significant increase in the number of smartphone users and owners among the African population. The Global System for Mobile Association (GSMA) forecasted the number of smartphone connections in SSA to reach 678 million by the end of 2025 with an adoption rate of 65% (5). However, in implementing mobile-based health interventions in low- and medium-income countries (LMIC), the social, economic, and cultural factors must be considered to make them more effective. Particularly, much attention has to be paid to rural areas where the majority of people are poor and lack formal education (6).

The current review aims to identify the existing mobile serious games and the challenges encountered in their design and use as persuasive tools for promoting healthy behavior change in rural Africa. It suggests design solutions for overcoming these challenges and how persuasive games can be optimized and/or tailored to suit the targeted rural African populations. The findings will guide persuasive digital game researchers, designers, and developers in understanding the challenges faced by users in rural African communities. Further, the idea is to inform the requirements needed when designing persuasive games targeting people in these underserved communities. A set of recommendations are finally outlined to develop digital serious games tailored to people in resource-constrained areas throughout the world other than rural African communities.

## Definition of key terms

### Health literacy

The term “health literacy” refers to the ability to access, understand, assess and apply information in a way that promotes, maintains and improves the health of individuals. However, the World Health Organization (WHO) defines it even more broadly as the ability of individuals to “gain access to, understand and use information in ways which promote and maintain good health” for themselves, their families and their communities (7). At the same time, health literacy, as a concept, includes many skills such as reading, writing, basic arithmetic, learning and speaking. The definition also includes skills related to computers, technologies and cell phones (8). Health literacy is a key component of public health, and improving it has become a major public health issue (9). Consumers' ability to search, find, appraise and use health information from the internet is known as an e-health literacy skill. E-health literacy includes a combined set of six basic skills: traditional literacy, health literacy, information literacy, scientific literacy, media literacy and computer literacy (10).

A multinational study on health literacy conducted in 14 countries in Sub-Saharan Africa between 2006 and 2015 showed an overall health literacy prevalence of 35.77% (11). But it should be notated that health literacy levels variated from country to country, from 8.51% in Niger to 63.89% in Namibia (11). E-health solutions, linked to the increasing penetration of mobile technology, contribute to overcoming health literacy challenges in Sub-Saharan Africa (12).

### Mobile serious games

According to Susi et al. (13), serious games have various definitions but share the same similar context: digital games whose goals go beyond pure entertainment. Serious games are “games that do not have entertainment, enjoyment or fun as their primary purpose” (14). The primary purposes of serious games can be but are not limited to, education, training, human resource management, and health improvement (14). Serious games include interactive computer applications, with or without a large hardware component, that provides the user with useful skills, knowledge, or attitudes, are demanding, entertaining to play, and engaging, according to (15). Designing serious games for non-literate users is a major challenge (16). Indeed, it requires a lot of testing and iterations during game development (16). Mobile serious games could be considered serious games designed and implemented to work more specifically on mobile phones (17).

## Digital development in Sub-Saharan Africa

SSA has experienced rapid growth in the use of mobile phones, computers, and internet access. According to a recent GSMA report, the SSA mobile phone market is expected to reach a compound annual growth rate of 4.6%—compared with 3% globally—between 2019 and 2025. This makes it one of the fastest-growing mobile phone subscribers in the world (18). The development of mobile telephony in SSA was quickly followed by the development of mobile health (aka m-health). These m-health applications concern the general population (prevention advice), patients (chronic diseases), health personnel (training, advice), the health information system (notification of cases of diseases under surveillance, alert), and research (questionnaire implementation). For example, the following are some of the most popular mobile applications developed the in SA region (19):

- Hello Doctor (South Africa): advice and medical assistance.
- Mobile Widewife (Nigeria): voice messages sent to pregnant women for follow-up pregnancy.
- M-Pedigree (Kenya): drug identification.
- My Healthline (Cameroon): answers to questions on sexuality, family planning and HIV/AIDS.
- mHero (Liberia): information on the virus outbreak Ebola.
- Djobi (Mali, Senegal): mobile application contributing to reducing infant mortality and kindergarten in Senegal and Mali through mutual health insurance. These first experiences have shown the interest that SSA has regarding the use of mobile telephony for health actions.

In the following section, we detail the method that has been followed to perform the literature review. In particular, the inclusion criteria and the databases that have been looked up are described.

## Methods

The inclusion criteria for the study selection were based on the research question, *what are the currently available mobile game interventions aimed to improve health literacy in Africa?* Referring to health literacy as “the ability to access, understand, evaluate, and apply health information” (20), we searched for reported mobile applications that promote “accessing,” “understanding,” “evaluation,” and “application” of health information to realize healthy behaviors and positive health outcomes (21). The interventions had to include at least one mobile technology-based delivery components such as smartphones, tablets or smartwatches, and intend to promote positive behavior change and lifestyle for improved health outcomes. Our study is performed according to the PRISMA protocol (22). A wide range of relevant studies, including articles and other publications published between 2011 and July 2021 in English, were included to identify mobile serious game interventions in the last 10 years in Africa. The authors chose 10 years for the search (2011–2021) to identify mobile serious games deployed in SSA because the continent has experienced exponential growth in access to mobile phones over the last decade (23).

### Database selection

To perform our literature review, PubMed, Scopus, Google Scholar and manual search were performed by utilizing the accompanying query terms.

### Keyword search

We performed our database search on 25 July 2021 using Boolean Operators (“AND,” “OR”). We used selected keywords to search the “titles, abstracts, and keywords” of articles related to the use of mobile games to improve health literacy, published in searched databases. The following combinations were used to perform this search: (mobile health^*^) AND (serious game^*^) AND (education and awareness) AND (Africa).

### Study selection

After performing the keyword search, we decided to limit our selection to English-language publications. Due to the rapid development of mobile technologies, we restricted our literature review to studies published between 2011 and 2021. No author restriction was imposed.

### Data extraction

We exported data related to the “title,” “abstract,” “keywords,” “author,” “publication dates,” and “country of origin” to a comma-separated-values (CSV) file. Then, we reviewed the full text of all publications according to the following criteria:

- studies focused on mobile serious games, education and awareness; otherwise, they were excluded;
- studies targeting public health issues in Africa;
- studies related to health literacy and mobile serious games in rural areas were included.

We carefully analyzed the publications according to the criteria mentioned above and the resulting selected data were converted to a CSV file format. We excluded studies that did not meet the above criteria.

## Results

### Included mobile-based serious games

**Figure 1:**
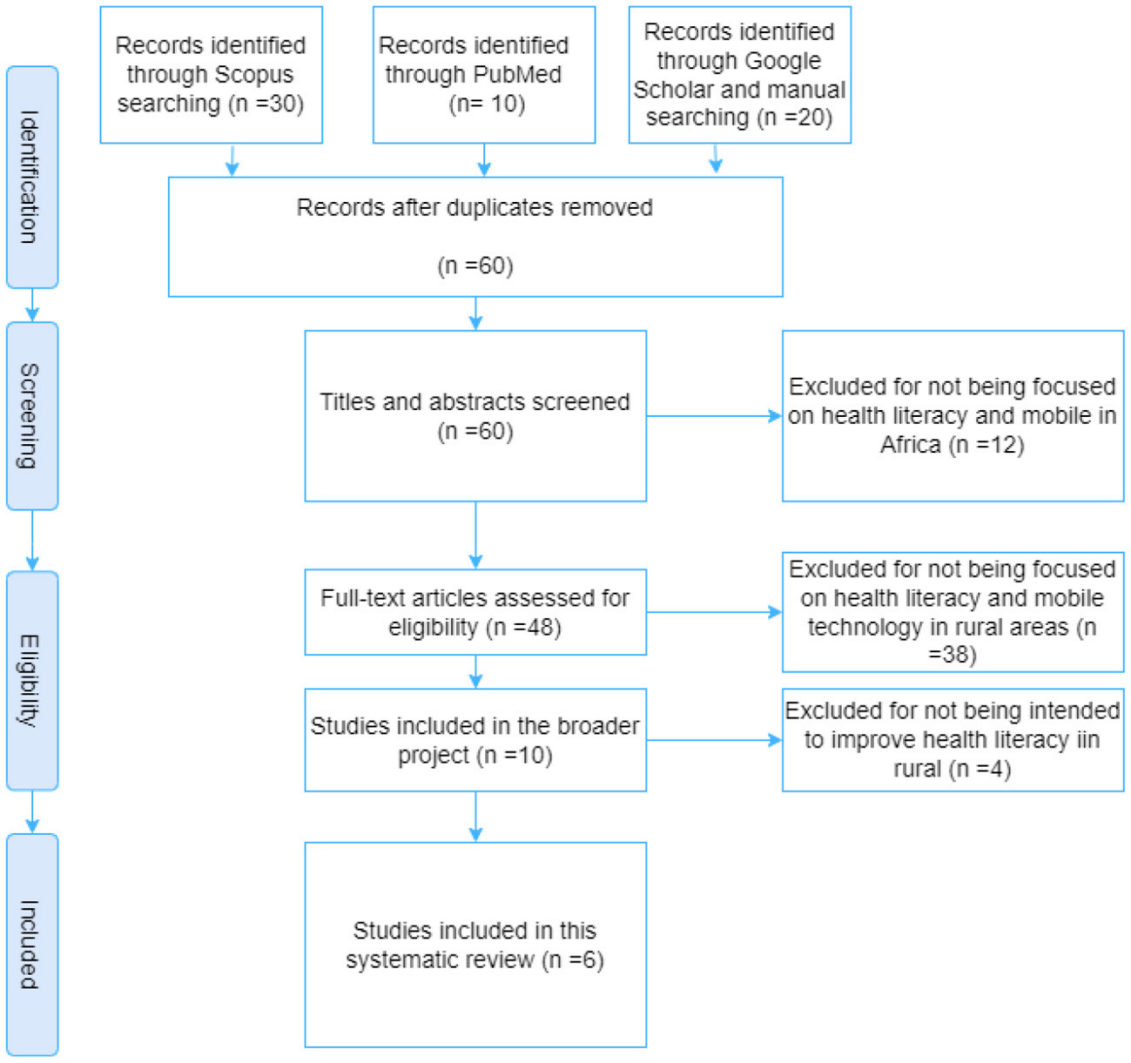
Flow diagram for the study selection process.

Figure 1 shows the selection process for the included articles in the literature review. Following our search using the keywords, 30 publications were found in the Scopus database, 10 in PubMed, and 20 in Google Scholar. No duplicates were found. During the title/abstract screening, we excluded 12 articles in total because they did not meet the criteria, leaving 48 for full-text screening. Upon completion of the full-text screening, 10 articles were selected. With further full-text screening, we found that only 6 articles were related to mobile serious games, education, and awareness of health information in rural Africa.

### General characteristics of interventions

As shown in Table 1, interventions included in this study were implemented in Nigeria (*n* = 2), Kenya (*n* = 2), Swaziland (*n* = 1), and Ghana (*n* = 1). Only two (*n* = 2) games, Tumaini and SwaziYolo, were assessed through a randomized controlled trial. The serious games targeted behavior change in HIV prevention among young people (*n* = 3), malaria preventive behaviors (*n* = 2) and Ebola prevention (*n* = 1). All the digital solutions were designed in the English language and support only Android devices. Moreover, all of these interventions required internet access.

**Table 1:** Mobile serious games interventions to promote health literacy.

**Game**	**Description of the game**	**Country**	**Characteristics of the technology**	**Outcomes**	**Challenges**
SwaziYolo	SwaziYolo, a smartphone game, is an interactive, educational narrative game in which the user plays the character of a young adult seeking love in Swaziland, making crucial decisions regarding relationships and sexual health (24). The game targets young adults (18–29 years)	Swaziland	Language of the game: English. The game is a 49 MB Android operating system. Internet is required	SwaziYolo game increased the intention to reduce sexual partnerships and increase the intention to know own and partner's HIV status.	-The high cost of facilitator-led group sessions and their poor record of translation from RCT to community setting (25).
My Jorley	Serious gaming for healthy sexual behavior, in Ghana and beyond (26). The game targets young people	Ghana	Language of the game: English. Android system. Internet is required	Target sexual behavior change among young people.	The culture of gaming was disregarded or seen as unserious in some contexts (27).
ChopUp Game (Ebola Strike Force)	Ebola Strike Force represents a paradigm shift in mobile gaming, empowering users not only to experience thrilling gameplay but also to learn about the Ebola virus in an interactive and immersive manner (28). The game targets all ages.	Nigeria	Language of the game: English. Android system. Internet is required	Over 700,000 users have played the ChopUp Game series (29).	Users have to pay N30 ($0.12) weekly to have access to the game (30).
Mosquito Smasher	Mosquito Smasher Game is a FUN game where you need to smash mosquitoes as much as you can. Every successful tap of a mosquito counts a point (31). The game targets all ages.	Nigeria	Language of the game: English. Android system. Internet is required	Increasing awareness of the disease.	High operating costs, with daily power, cuts the norm
Mosquito Hood	Mosquito Hood is a fast-paced 2D casual game that aims to save Africa from the mosquito-borne disease Malaria by spreading real nets across the continent. Players earn points for advancing through sprite levels and for completing the game's objective. The game is meant for all ages.	Kenya	Language of the game: English. Android system. Internet is required	1,400 households in high-risk malaria zones in rural Kenya received insecticide-treated mosquito nets after playing the Mosquito Hood game (32).	A realistic assessment of Mosquito Hood is needed (32).
Tumaini	A narrative-based smartphone game designed to help prevent HIV among young Africans (33). The game targets 11–14 years.	Kenya	Language of the game: English. Internet is required Android system	Using the Tumaini game has shown significant gains in sexual health-related knowledge and self-efficacy (33).	The full functionality of the game should not require internet access (33).

## Discussion

### Principal findings

Our findings suggest that mobile serious games could enhance access to health information and therefore contribute to healthy behavior change among young people (11–14 years) and adults (18–29 years) (24, 33). A randomized controlled trial of the SwaziYolo game showed an increased intention to reduce sexual partnerships, and an increased intention to know one's partner's HIV status among young adults (18–29 years) (24). The assessment of the Tumaini game also showed significant gains in sexual health-related knowledge and self-efficacy among 11–14 years (33). During malaria prevention and sensitization campaigns, serious games could be used as motivational tools to help reach out to populations living in remote areas. Over 1,400 households in high-risk malaria zones in rural Kenya received insecticide-treated mosquito nets after playing the Mosquito Hood game (32).

However, due to limited resources in rural areas, implementing sustainable mobile-based healthcare interventions is a great challenge. ChopUp Game (Ebola Strike Force) users in Nigeria for instance, had to pay N30 ($0.12) weekly to have access to the game in 2015 (30). Knowing that among those living below the $1.90 poverty line in 2019, 84.6% lived in rural Nigeria (34). Costs for accessing the games can be unaffordable for these rural populations. Another challenge is access to electricity. Indeed, only 24.6% of Nigeria's population had access to electricity (35). Despite the nearly doubling of mobile technology penetration in Africa, internet connection in rural regions remains limited. In addition, understanding the social, economic, and cultural context in which the technology will be implemented is essential for its success. As noted during the deployment of My Jorley in Ghana: “games can be disregarded or seen as unserious in some cultures” (27). We observe that all included games in our study were designed in the English language and required an internet connection. Implementing games which require internet access can be inaccessible for rural populations where the internet is usually inaccessible or intermittent (33). Considering also that one-third of the people in SSA aged 15 and above were unable to read and write in 2017 (36), designing a technology with Graphical User Interface (GUI) which requires reading and writing could be inaccessible for low-literate users.

### Comparison with serious games-based healthcare interventions in other regions

Serious games mHealth interventions have a great potential to reach low-literate people in LMICs countries. MANTRA project in Nepal for instance is a good example (37). The project aimed to provide educational content as learning through gaming for non-literate and low-literate users and does not require a smartphone to use and play it. Therefore, the game was designed without text (for those unable to read). A tutorial for drag and drop (for those unfamiliar with smartphones) was also provided. Inclusive digital solutions are those designed with all the users in mind, focusing on their different needs and contexts (38). In this frame, the GADSA project aimed to improve antibiotic stewardship amongst surgeons in Nigeria through a gamified decision support app integrating the WHO and Stanford. antimicrobial guidelines (39). It was designed to cater to various users in a unique partnership between academic researchers, graphic designers, software developers, infection control specialists and healthcare professionals (39).

Behavior change games are serious games designed to promote attitude and behavior change (40). They have the same purpose and characteristics as what is commonly known as persuasive games (3). They fall under the umbrella of persuasive technologies, which are interactive digital tools intended to alter attitudes or behaviors by making desired outcomes simpler to attain (3).

In terms of targeted diseases, our literature review found three games that promoted HIV prevention behavior change among young people. The games include SwaziYolo aiming at reducing multi-sexual partnerships HIV (24), Tumaini for increasing sexual health-related knowledge (33), and My Jorley for improving young people's knowledge about HIV and fostering and behavior (26). Moreover, we found three other games aimed at increasing malaria awareness in Africa. Mosquito Hood increased the awareness of 14,000 Kenyan households on malaria preventive behaviors (32). Mosquito Smasher increased the awareness of Nigerians. The Ebola Strike Force game has generated a great deal of interest in malaria prevention measures in Nigeria; however, Mosquito Hood and Ebola Strike are yet to be evaluated empirically (29). Notably, none of the reviewed mobile serious games addressed maternal and newborn health in rural Africa, despite the fact that SSA accounted for almost two-thirds (196,000) of maternal fatalities (41). Serious games such as MANTRA could contribute to improving knowledge and awareness of maternal health and neonatal health, especially among women in rural areas of SSA.

## Recommendations to address challenges

Mobile serious games could enhance access to health information and therefore contribute to healthy behavior change. Although Ndulue and Orji (42) provided some recommendations to tackle some of the challenges (see Table 2), many remain unaddressed.

**Table 2:** Summary of recommendations for serious mobile game design for rural African areas (42).

**Challenge**	**Recommendation**
Language diversity	Use of symbols.
Internet issues	Minimal internet dependence, minimal bandwidth usage, and a minimum of one update per month.
Energy consumption and electricity	Minimal animations and graphics, less background activity.
Memory space and CPU power	Minimal animations and graphics, minimal game size, minimal data storage on phone.
Low literacy	Use of audio tools for passing information, and use of culturally meaningful symbols.
Game concepts, cost and culture	Proper research on target population culture, developing for individual ethnic groups.

### Understanding local environments

In rural communities, healthcare workers encounter numerous challenges.

Due to a lack of funding, technical resources such as computers and the internet are restricted and unreliable.

In addition, there is a severe shortage of infrastructure for storing electronic data.

The budget and number of technical staff for managing mobile technologies are constrained.

Therefore, designed technology and implementation and management expenses must account for these constraints.

Changes in government, potential civil unrest, staff turnover, and environmental circumstances such as floods or natural disasters could also affect the local environment during the duration of the project.

### Time and cost

As there are fewer medical facilities in rural areas, they are frequently overcrowded. For the technology to be successful, training and installation must be completed in a timely manner. Patients in rural areas may have restricted financial resources to subscribe to games if subscription fees are greater. Users of ChopUp Game (Ebola Strike Force) were required to pay N30 ($0.12) per week to access the game (30), which can be a great challenge for rural populations due to the low per-capital income and the fact that many live below the poverty line.

### Diversity of culture

SSA has a large and diverse population whose customs and traditions may vary with different languages. Thus, the design may need to focus on a smaller group of the population if there are cultural adaptations and to tailor the incentives for behavior change.

### Low literacy

It is known that the illiteracy rate is high in SSA and rural areas in particular. This low level of literacy is another challenge for patients when it comes to writing or reading health-related information. Mobile-based games must consider voice, graphics, and video-based GUIs (43).

## Limitations

The main limitation of our literature review is that it was based only on three databases: PubMed, Scopus, and Google Scholar. Consequently, we were unable to include additional studies that might have been indexed by other databases or available through the gray literature. Another limitation is that our keywords are limited in scope. For example, we only used the term “mobile health” in our search string without including its other variations such as “mHealth,” “m-Health,” etc. Future work should aim to address these limitations. This may provide access to more academic papers and projects carried out by non-governmental organizations (NGOs).

## Conclusion and suggestions for further research

Our literature review enabled the identification of six studies about the use of mobile games to promote health literacy in rural Africa. The findings suggest that mobile serious games could improve access to health information and, as a result, promote healthy behavior modification among adolescents and adults. Serious games could also be used as motivational tools to reach out to remote populations for health education and promotion. However, we did not find a game designed with functionalities and content in SSA local language. This could limit the accessibility to users with limited literacy. For software designers to develop inclusive mhealth solutions, a framework that may act as a guide will be required. Integrating local languages into the rural African context would be crucial. Future studies will investigate and design a relevant framework to assist the introduction of more inclusive mobile technology in rural Africa.

## Data availability statement

The original contributions presented in the study are included in the article/supplementary material, further inquiries can be directed to the corresponding author.

## Author contributions

IO and GD conceived the study. GD, BS, KO, and RB validated the methodological approach and the research equations. IO wrote the first draft of the manuscript. All the authors analyzed the results, participated in the final review of the manuscript, and corrected and approved the manuscript for submission.

## Funding

The study was supported by the PATIENT-COVID-19 project.

## Conflict of interest

The authors declare that the research was conducted in the absence of any commercial or financial relationships that could be construed as a potential conflict of interest.

## Publisher's note

All claims expressed in this article are solely those of the authors and do not necessarily represent those of their affiliated organizations, or those of the publisher, the editors and the reviewers. Any product that may be evaluated in this article, or claim that may be made by its manufacturer, is not guaranteed or endorsed by the publisher.
